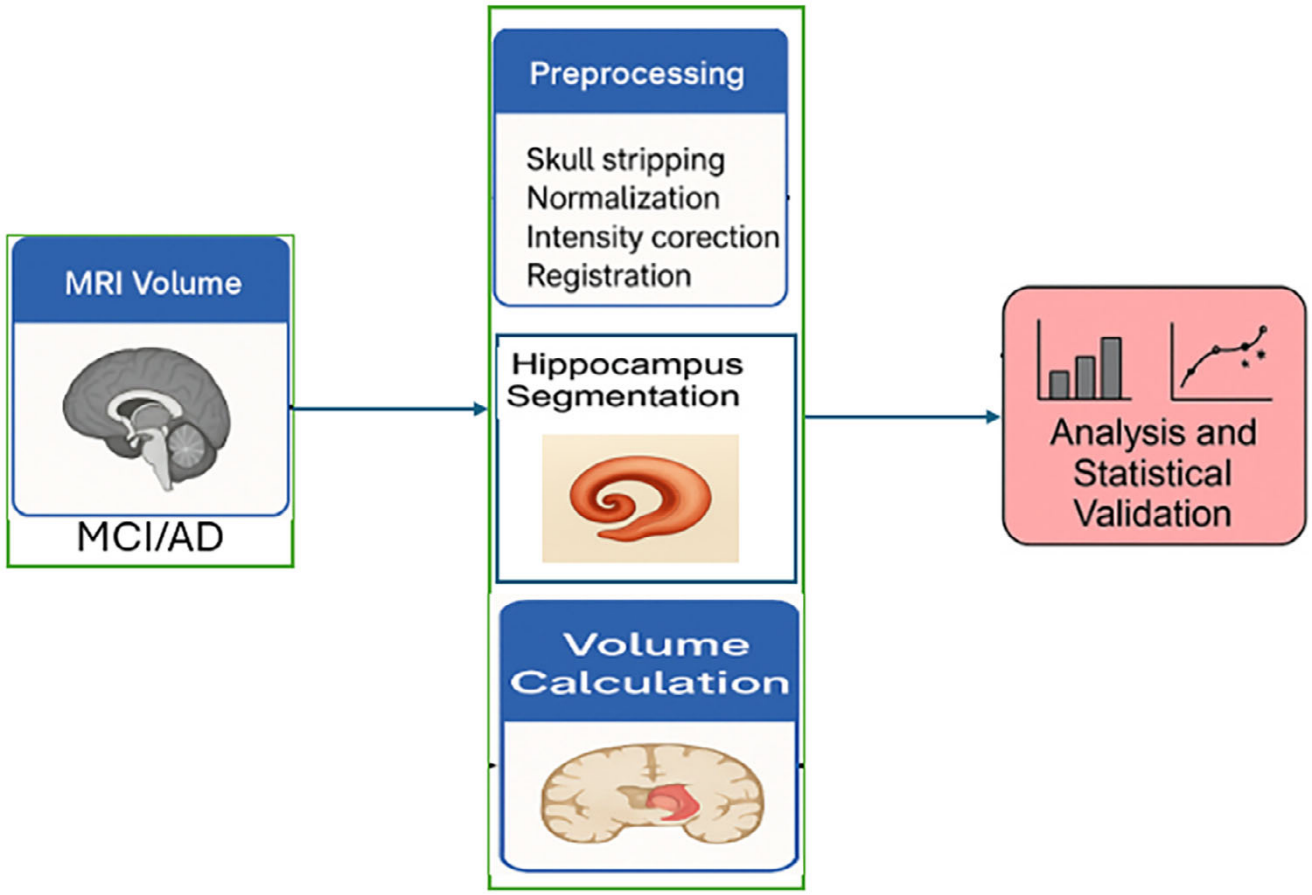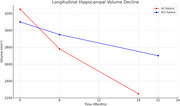# Identifying High‐Risk Patients Through Longitudinal Hippocampal Atrophy Patterns in MCI and AD

**DOI:** 10.1002/alz70861_108226

**Published:** 2025-12-23

**Authors:** Abraham Varghese, Kesavadas Chandrasekharan, Kannan Balakrishnan

**Affiliations:** ^1^ University of Technology and Applied Sciences, Alkhuwair, Muscat Oman; ^2^ Sree Chitra Tirunal Institute for Medical Sciences and Technology, Trivandrum, Kerala India; ^3^ Emeritus Professor, Department of Computer Applications, Cochin university of Science and Technology, Cochin, Kerala India

## Abstract

**Background:**

The hippocampus is among the earliest regions affected in Alzheimer’s disease (AD) and is critical for tracking disease progression from mild cognitive impairment (MCI). Detecting accelerated hippocampal atrophy could help identify individuals at high risk of conversion to AD. While tools like FreeSurfer are widely used, they are computationally intensive. Recent advancements in deep learning–based segmentation with attention mechanisms offer scalable, automated, and interpretable alternatives. This study aims to (1) demonstrate early evidence of differential atrophy patterns in AD and MCI using a lightweight method, (2) develop a pipeline for large‐scale analysis of 150 patients per group, and (3) integrate deep learning segmentation with statistical modeling for high‐sensitivity detection.

**Methods:**

Longitudinal T1‐weighted MRI scans from the ADNI database were analyzed for AD and MCI subjects, each with three time points spanning 6 to 24 months. Preprocessing included skull stripping, intensity correction, normalization, and registration. Hippocampal segmentation was performed using a deep learning model with attention mechanisms. Results were validated against FreeSurfer using Dice similarity scores. Volumes were computed across time points, and statistical evaluation using repeated‐measures ANOVA and linear mixed‐effects modeling is planned. An overview of the methodology is shown in Figure 1.

**Results:**

Sample results from one AD and one MCI patient demonstrate distinct progression trends.

AD Patient: Volume declined from 3,250 mm³ (baseline) to 2,250 mm³ (18 months), a ∼30% reduction. MCI Patient: Volume declined from 3,100 mm³ to 2,700 mm³ (21 months), a ∼13% reduction. The Figure 2 shows the hippocampal atrophy of MCI and AD categories of a representaive participant.

These findings suggest faster hippocampal atrophy in AD. Full cohort analysis is underway to validate this trend statistically.

**Conclusion:**

This pilot analysis supports the use of rapid segmentation techniques for early risk detection in MCI and AD. The marked difference in atrophy rates between groups underscores the need for large‐scale validation using deep learning and statistical analysis. An automated tool for hippocampal volume tracking could be instrumental in both research and future clinical practice.